# Pathogenic potential and antimicrobial resistance of *Staphylococcus pseudintermedius* isolated from human and animals

**DOI:** 10.1007/s12223-022-01007-x

**Published:** 2022-10-12

**Authors:** Paulina Glajzner, Eligia M. Szewczyk, Magdalena Szemraj

**Affiliations:** Department of Pharmaceutical Microbiology and Microbiological Diagnostics, Medical University of Lodz, ul. Muszyńskiego 1, 90-001 Lodz, Poland

**Keywords:** Bacterial adhesions, Staphylococcal infections, Zoonotic infections, *Staphylococcus*, Methicillin resistance

## Abstract

Crossing of interspecies barriers by microorganisms is observed. In recent years, *Staphylococcus pseudintermedius*—a species formerly thought to be animal—has also been isolated from human clinical materials. Many virulence factors are responsible for the colonization, which is the first step an infection, of the new host organism. We analyzed the factors influencing this colonization as well as susceptibility to antibiotics in fourteen *S. pseudintermedius* strains isolated from clinical cases from humans and animals. The occurrence of genes responsible for binding elastin, fibronectin, and fibrinogen and some phenotypic features, although different between strains, is comparable in both groups. However, the animal isolates had more genes coding for virulence factors. All isolates tested had the exfoliating toxin gene and the leukotoxin determining genes, but only the human strains had enterotoxin genes. The assessment of antibiotic resistance of strains of both groups indicates their broad resistance to antibiotics commonly used in veterinary medicine. Antibiotic resistance was more common among animal isolates. The multilocus sequence typing analysis of the studied strains was performed. The results indicated a large diversity of the *S. pseudintermedius* population in both studied groups of strains. Equipped with important virulence factors, they showed the ability to infect animals and humans. The clonal differentiation of the methicillin-susceptible strains and the multidrug resistance of the strains of both studied groups should be emphasized. The considerable genetic diversity of strains from a limited geographical area indicates the processes of change taking place within this species. Thus, careful observation of the ongoing process of variation is necessary, as they may lead to the selection of *S. pseudintermedius*, which will pose a significant threat to humans.

**Supplementary Information:**

The online version contains supplementary material available at 10.1007/s12223-022-01007-x.

## Introduction

In recent years, viruses and bacteria have been crossing interspecies barriers more and more often. Adaptation to different hosts allows them to spread effectively (Gortazar et al. 2014). Pathogens that until recently were considered to be typically animal, causing only diseases in animals or constituting their microbiota, can now also cause human infections, including life-threatening ones (Van Hoovels et al. 2006; Chrobak-Chmiel et al. 2018; Gonzalez-Martin et al. 2020).

*Staphylococcus pseudintermediu*s is a coagulase-positive species of staphylococci, commonly colonizing the skin and mucous membranes of animals, most often dogs and cats (Bardiau et al. 2013; Pitchenin et al. 2017; Chrobak-Chmiel et al. 2018). It has also been proven that these bacteria are transmitted to humans in contact with animals (Phumthanakorn et al. 2017). In animals, *S. pseudintermedius* is often isolated from wounds, ears, bones, or postoperative abscesses (Abouelkhair et al. 2018; Maali et al. 2018). *S. pseudintermedius* is also found in human infections. The most common are wound infections, mainly after a dog bite, soft tissue infections, otitis externa, and sinusitis. Severe infections such as septic arthritis, nosocomial pneumonia, endocarditis, and bacteremia have also been reported. Contact between an infected animal and person is indicated as crucial in these infections (Van Hoovels et al. 2006; Chrobak et al. 2011; Bannoehr and Guardabassi 2012; Viau et al. 2015; Kmieciak and Szewczyk 2018; Maali et al. 2018; Phumthanakorn et al. 2018). Therapeutic management of *S. pseudintermedius* infections has become challenging due to the emergence of methicillin-resistant strains (Carroll et al. 2021).

The pathogenesis of *S. pseudintermedius* infections follows the stages typical of staphylococci. Adhesion begins the colonization of an organism. The virulence factors responsible for this process are surface adhesins, which recognize host extracellular matrix adhesives (MSCRAMM) (Crawford et al. 2016; Phumthanakorn et al. 2017). Their presence supports the invasion and active colonization of host epithelial cells and thus develops infection (Zuniga et al. 2015; Maali et al. 2018). The production of many enzymes, including coagulase, and the ability to form biofilms determine the multiplication of bacteria and, consequently, the development of infection. It has been shown that some strains can also produce toxins (Garbacz et al. 2013; Pitchenin et al. 2017). The presence of these characteristics in strains isolated from human clinical material indicates a risk that should be considered. The spread of clones and their resistance requires constant monitoring.

We studied *S. pseudintermedius* strains isolated in municipal laboratories from swabs and pus from humans and companion animals. They were the etiological cause of existing infections. Potential factors influencing colonization and spread in the infected organism and susceptibility to antibiotics were investigated. Clonal relationships between the strains were analyzed by comparing the seven housekeeping gene sequences (MLST, multilocus sequence typing analysis) to evaluate the diversity of the isolated strain populations.

## Materials and methods

### Growth conditions of the tested strains

Fourteen *S. pseudintermedius* strains isolated from wounds, ears, and abdominal cavity in the hospital laboratories (seven human strains) and veterinary laboratories (seven strains from accompanying animals) in Lodz city, Poland, were used.

Strains selected for this study were initially identified as *S. pseudintermedius* and *S. intermedius* by MALDI-TOF MS. However, genetic identification based on the sequence of the *nuc* gene showed that all tested strains belonged to the *S. pseudintermedius* species (Sasaki et al. 2010). The strains were isolated and propagated on Columbia Agar and for antibiotic susceptibility testing on Mueller–Hinton media. DNA isolation was performed after an overnight cultivation of the isolates in the Brain Heart Infusion broth.

### Phenotypic features

The ability to produce coagulase, lipase, lecithinase, caseinase, and gelatinase CF aggregating factor (clumping factor) test was determined according to the methods described earlier (Freney et al. 1999; Chakraborty et al. 2011; Haddad et al. 2018). *Staphylococcus aureus* ATCC 25923 was used as the control.

Susceptibility to antibiotics was tested by the disc diffusion method according to European Committee on Antimicrobial Susceptibility Testing (EUCAST) guidelines for human strains and to Clinical and Laboratory Standards Institute (CLSI) guidelines for animal strains (CLSI 2015; EUCAST 2019). In the study, 14 antibiotics recommended in the treatment of staphylococcal human and animal infections were used: penicillins (oxacillin, benzylpenicillin), fluoroquinolones group (ciprofloxacin, norfloxacin), aminoglycosides (gentamicin), lincosamides and macrolides group (clindamycin, erythromycin), tetracyclines group (tetracycline, tigecycline), quinupristin/dalfopristin, trimethoprim/sulfamethoxazole, linezolid, fusidic acid, and rifampicin. Due to the lack of antibiotic breakpoints in CLSI VET01S other than clindamycin and tetracyclines for staphylococcal strains isolated from animals, human clinical values with EUCAST were used in this study.

The sensitivity to oxacillin was determined to detect methicillin-resistant phenotype (MRSP). *Staphylococcus aureus* ATCC 29213 was used as a quality control strain. For oxacillin-resistant strains, the MIC values (minimum inhibitory concentration) for vancomycin and daptomycin were determined. Results were interpreted according to EUCAST (EUCAST 2019).

### Genotypic features

Bacterial DNA isolation was performed according to the Genomic Mini AX Staphylococcus Gravity protocol (A&ABiotechnology 2020).

#### Detection of genes responsible for the colonization and development of infections

Table 1 shows the genes searched in this study. Genes with sequences similar or identical to those found in *S. aureus* are found in various species of *Staphylococcus*. This homology allows their detection using primers designed for this species. These sequences were also used in this study. Table 1 presents the origin of the searched sequences and the size of the amplicons. The bacterial species for which the presence of the genes was tested based on the proposed sequence were also listed.

**Table 1:** Characteristics of the studied genes

Coded trait	The name of gene	The origin of the genetic sequence	Amplicon size	Bacterial species for which the presented sequence was used	References
Genes responsible for matrix protein binding					
Elastin-binding protein	*ebpS*	*S. pseudintermedius*	1040 bp	*S. pseudintermedius*	Kmieciak and Szewczyk (2018)
Protein binding fibrinogen, fibronectin, and elastin	*spsO*	*S. pseudintermedius*	696 bp	*S. pseudintermedius*	Phumthanakorn et al. (2017)
Immunoglobulin binding protein A	*spsP*	*S. pseudintermedius*	366 bp	*S. pseudintermedius*	
	*spsQ*	*S. pseudintermedius*	217 bp	*S. pseudintermedius*	
Protein binding fibrinogen, fibronectin, and elastin	*spsL*	*S. pseudintermedius*	201 bp	*S. pseudintermedius*	
	*spsD*	*S. pseudintermedius*	528 bp	*S. pseudintermedius*	
Binding of fibrinogen	*spsE*	*S. pseudintermedius*	1600pz	*S. pseudintermedius*	Kmieciak and Szewczyk (2018)
Binding of fibrinogen	*fib*	*S. aureus*	404 bp	*S. aureus*, *S. schleiferi* subsp. *coagulans*, *S. hyicus*, *S. intermedius*	Tristan et al. (2003); Zuniga et al. (2015)
Binding of fibronectin	*fnbB*	*S. aureus*	524 bp	*S. aureus*, *S. schleiferi* subsp. *coagulans*, *S. hyicus*, *S. intermedius*	
	*fnbA*	*S. aureus*	1280 bp	*S. aureus*, *S. schleiferi* subsp. *coagulans*, *S. hyicus*, *S. intermedius*	Zuniga et al. (2015)
Collagen binding	*cna*	*S. aureus*	423 bp	*S. aureus*, *S. schleiferi* subsp. *coagulans*, *S. hyicus*, *S. intermedius*	Tristan et al. (2003); Zuniga et al. (2015)
Laminin binding	*eno*	*S. aureus*	302 bp	*S. aureus*, *S. schleiferi* subsp. *coagulans*, *S. hyicus*, *S. intermedius*	
Sialoprotein binding	*bbp*	*S. aureus*	575 bp	*S. aureus*	Wisniewska et al. (2014); Thompson and Brown (2017); Firoozeh et al. (2020); Cavalcante et al. (2021)
Production of coagulase	*coa*	*S. pseudintermedius*	1500 bp	*S. pseudintermedius*	Sewid et al. (2018)
Genes responsible for the production of biofilm					
Production of biofilm	*icaC*	*S. aureus*	1100 bp	*S. pseudintermedius*, *S. epidermidis*, *S. aureus*	Ghasemian et al. (2015); Crawford et al. (2016); Piechota et al. (2018); Parastan et al. (2020)
	*icaD*	*S. pseudintermedius*	166 bp	*S. pseudintermedius*	Casagrande Proietti et al. (2015); Meroni et al. (2019)
	*bap*	*S. aureus*	971 bp	*S. auricularis*, *S. capitis* subsp. *capitis*, *S. chromogenes*, *S. cohnii* subsp*. cohnii*, *S. epidermidis*, *S. haemolyticus*, *S. hominis* subsp. *hominis*, *S. lugdunensis*, *S. saprophyticus*, *S. schleiferi* subsp. *coagulans*, *S. warneri*, *S. xylosus*, *S. aureus*, *S. intermedius*, *S. hyicus*	Cucarella et al. (2001); Vancraeynest et al. (2004); Salaberry et al. (2015); Zuniga et al. (2015)
Genes responsible for the production of toxins					
Production of enterotoxins	*sea*	*S. aureus*	127 bp	*S. aureus*, *S. simulans*, *S. warneri*, *S. sciuri*, *S. haemolyticus*, *S. lentus*, *S. saprophyticus*, *S. epidermidis*, *S. xylosus*, *S. cohnii*, *S. pasteuri*	Çetin and Tuncer (2016); Lange et al. (2020); Szemraj et al. (2020)
	*seb*	*S. aureus*	477 bp	*S. aureus*, *S. intermedius*, *S. pseudintermedius*, *S. haemolyticus*, *S. epidermidis*, *S. warneri*, *S. saprophyticus*, *S. simulans*, *S. xylosus*, *S. cohnii*, *S. chromogenes*, *S. hominis*, *S. capitis*	Garbacz et al. (2013); Rall et al. (2014); Mello et al. (2016); Saad et al. (2017)
	*sec*	*S. aureus*	271 bp	*S. aureus*, *S. chromogenes*, *S. hyicus*, *S. xylosus*, *S. sciuri*, *S. epidermidis*, *S. intermedius*, *S. pseudintermedius*	Becker et al. (2001); Park et al. (2011); Garbacz et al. (2013); Piechota et al. (2014); Bergot et al. (2018); Lange et al. (2020)
	*sed*	*S. aureus*	319 bp	*S. aureus*, *S. epidermidis*, *S. xylosus*, *S. pseudintermedius*	Garbacz et al. (2013); Tanabe et al. (2013); Phumthanakorn et al. (2018); Piechota et al. (2018)
	*see*	*S. aureus*	178 bp	*S. aureus*, *S. pseudintermedius*, *S. chromogenes*, *S. epidermidis*, *S. saprophyticus*, *S. haemolyticus*	Garbacz et al. (2013); Phumthanakorn et al. (2018); Lange et al. (2020); Nasaj et al. (2020)
Production of exfoliative toxins	*exiA*	*S. pseudintermedius*	455 bp	*S. pseudintermedius*	Melter et al. (2017)
	*exiB*	*S. pseudintermedius*	381 bp	*S. pseudintermedius*	
	*siet*	*S. aureus*, *S. intermedius*	359 bp	*S. pseudintermedius*, *S. intermedius*, *S. aureus*, *S. epidermidis*, *S. haemolyticus*, *S. saprophyticus*, *S. sciuri*, *S. warneri*	Lautz et al. (2006); Ruzauskas et al. (2016); Meroni et al. (2019); Hritcu et al. (2020)
Production of Luk-SF leukotoxin	*LukS*	*S. pseudintermedius*	868 bp	*S. pseudintermedius*	Maali et al. (2018)
	*LukF*	*S. pseudintermedius*	926 bp	*S. pseudintermedius*	
Production of Panton-Valentine leukotoxin	*pvl*	*S. aureus*	433 bp	*S. pseudintermedius*, *S. aureus*, *S. schleiferi*	Ishihara et al. (2010); Ziasistani et al. (2019)
Genes responsible for the production of lipolytic enzymes					
Lipase production	*lip*	*S. pseudintermedius*	1601 bp	*S. pseudintermedius*	Kmieciak and Szewczyk (2018)

#### Detection of the *mecA* and *blaZ* genes

The *mecA* and *blaZ* genes were detected to confirm methicillin and penicillin resistance. The genes were detected using the primers and reaction conditions previously described (Kang et al. 2014).

PCR reactions were performed on a Professional Basic Gradient Thermal Cycler (Biometra). The reaction products were separated by electrophoresis on a 1% agarose gel stained with Midori Green DNA stain (NIPPON Genetics EUROPE, Germany) in TAE buffer for 60 min at 70 V and visualized under ultraviolet light.

#### Multilocus sequence typing (MLST) analysis

The genetic diversity of the investigated *S. pseudintermedius* isolates was determined by the MLST method using seven loci (*ack*, *cpn*60, *fdh*, *purA*, *pta*, *sar*, *tuf*) (Solyman et al. 2013). PCR products were sequenced (Genomed, Poland).

The sequences were analyzed and compared with known sequences in the PubMLST database (http://pubmlst.org/). The identified alleles of the seven genes of each strain were used to assign a specific sequence type (ST). All isolates were tested for association with the European sequence types reported in the MLST database. For this purpose, the geoBURST 1.2.1 program was used.

The DNA sequences generated in this study were deposited in GenBank, accession no. for the *ack* gene, OL378202–OL378215; for the *cpn*60 gene, OL378216–OL378229; for the *fdh* gene, OL378230–OL378243; for the *purA* gene, OL378258–OL378271; for the *pta* gene, OL378244–OL378257; for the *sar* gene, OL378272–OL378285; and for the *tuf* gene, OL372245–OL372258.

The sequences of the primers used in the genetic research are posted in Online Resource 1 in Table S1 (file ESM_1).

## Results

All the tested strains were coagulase positive, but the clumping factor (CF) tests were positive only in four human isolates. Proteases were found in both human and animal strains. Only one animal strain produced lecithinase. It was the one with the highest resistance and collected the most significant number of sought genes (Table 2).

**Table 2:** Phenotypic and genotypic features in the tested strains

Strain no.	Phenotypic features				Presence of the studied genes encoding			Resistance genes	Resistance phenotype
	CF	GEL	CAS	LEC	Enterotoxins	Exfoliative toxins	Host’s matrix proteins binding		
Human strains									
SPL1	+	−	+	−	-	*siet*	*ebpS*, *spsO*, *spsL*, *spsE*, *eno*, *coa*, *LukS*, *LukF*, *icaD*, *lip*	*blaZ*	PEN, TE
SPL2	−	−	+	−	*sed*	*siet*	*ebpS*, *spsL*, *spsE*, *eno*, *coa*, *LukS*, *LukF*, *icaD*, *lip*	*blaZ*	PEN, ERY, CLI
SPL3	−	−	+	−	-	*siet, exiA*	*ebpS*, *spsL*, *spsE*, *eno*, *coa*, *LukS*, *LukF*, *icaD*, *lip*	-	-
SPL4	−	+	+	−	*sec*	*siet, exiA*	*ebpS*, *spsO*, *spsL*, *spsE*, *eno*, *coa*, *LukS*, *LukF*, *icaD*, *lip*	*blaZ*	PEN
SPL5	+	−	+	−	-	*siet*	*ebpS*, *spsO*, *spsL*, *spsE*, *coa*, *LukS*, *LukF*, *icaD*, *lip*	*blaZ*	PEN, TE
SPL6	+	−	+	−	*sec*	*siet*	*ebpS*, *spsL*, *spsE*, *eno*, *coa*, *LukS*, *LukF*, *icaD*, *lip*	-	-
SPL7	+	−	+	−	*sec, sed*	*siet, exiB*	*ebpS*, *spsL*, *spsE*, *eno*, *coa*, *LukS*, *LukF*, *icaD*, *lip*	-	-

Animal strains									
SPZ1	−	+	+	−	-	*siet*	*ebpS*, *spsL*, *spsE*, *eno*, *coa*, *LukS*, *LukF*, *icaD*, *lip*	*blaZ*	PEN
SPZ2	−	−	+	−	-	*siet, exiB*	*ebpS*, *spsO*, *spsP*, *spsQ*, *spsL*, *spsE*, *eno*, *coa*, *LukS*, *LukF*, *icaD*, *lip*	*blaZ*	PEN, ERY, CLI, TE, NOR, CIP
SPZ3	−	+	+	−	-	*siet*	*ebpS*, *spsP*, *spsQ*, *spsL*, *spsE*, *eno*, *coa*, *LukS*, *LukF*, *icaD*, *lip*	*blaZ*	PEN, ERY, CLI, GEN, TE, NOR, CIP
SPZ4	−	+	+	+	-	*siet*	*ebpS*, *spsP*, *spsQ*, *spsL*, *spsE*, *fnbA*, *eno*, *coa*, *LukS*, *LukF*, *icaD*, *lip*	*mecA, blaZ*	PEN, OXC, ERY, CLI, GEN, TE, NOR, CIP, SXT
SPZ5	−	+	+	−	-	*siet*	*ebpS*, *spsL*, *spsE*, *eno*, *coa*, *LukS*, *LukF*, *icaD*, *lip*	*blaZ*	PEN, ERY, CLI, TE
SPZ6	−	−	+	−	−	*siet*	*ebpS*, *spsL*, *spsE*, *eno*, *coa*, *LukS*, *LukF*, *icaD*, *lip*	*blaZ*	PEN, ERY, CLI
SPZ7	−	+	+	−	-	*siet*	*ebpS*, *spsP*, *spsQ*, *spsL*, *spsE*, *fnbA*, *eno*, *coa*, *LukS*, *LukF*, *icaD*, *lip*	*mecA, blaZ*	PEN, OXC, ERY, CLI, GEN, TE, NOR, CIP, SXT

All strains produced lipase and were susceptible to rifampicin, fusidic acid, linezolid, and tigecycline

*CF* clumping factor, *GEL* gelatinase, *CAS* caseinase, *LEC* lecithinase, *PEN* penicillin G, *OXC* oxacillin, *ERY* erythromycin, *CLI* clindamycin, *GEN* gentamycin, *TE* tetracycline, *NOR* norfloxacin, *CIP* ciprofloxacin, *SXT* sulfamethoxazole/trimethoprim, “+” positive,“−” negative

Antibiotic resistance was more common among animal isolates. Both methicillin-resistant and methicillin-susceptible isolates were resistant to many classes of antibiotics. In addition to penicillin resistance, among the strains tested, the most common was resistance to erythromycin and clindamycin.

Only two of the 14 isolates tested were phenotypically resistant to methicillin. Genetic studies confirmed the presence of the *mecA* gene only for these strains. Only methicillin-resistant isolates (MRSP) showed resistance to sulfamethoxazole with trimethoprim. The *blaZ* gene was detected in 11 strains, consistent with the penicillin resistance phenotype. There was no resistance to the following antibiotics: rifampicin, linezolid and tigecycline, quinupristin/dalfopristin, and fusidic acid, used in severe infections. All strains were susceptible to vancomycin and daptomycin. The results of susceptibility testing of human and animal strains to antibiotics are presented in Table 2.

All tested isolates had essential genes allowing for efficient attachment to host tissues by binding elastin, fibronectin, and fibrinogen (*ebpS*, *spsL*, *spsE*, *coa*). Some of them had accumulated additional genes conditioning the nonspecific binding of immunoglobulins (*spsP*, *spsO*) and of fibronectin (*fnbA*). The presence of the highest number of genes necessary for adhesion to the cell matrix was accompanied by an antibiotic resistance phenotype (SPZ2, SPZ3, SPZ4, SPL6 isolates). All tested strains possessed the *icaD* gene, one of the three sought genes related to the biofilm production capacity. Only in human isolates enterotoxin genes (*sed*, *sec*) were detected. All tested isolates had the *siet* gene encoding the exfoliating toxin and the genes determining the production of leukotoxin and lipase (*LukS*, *LukF*, *lip*). However, none of the tested strains carried the *pvl* gene.

The following genes were not found in the studied isolates: *pvl* responsible for the production of Panton-Valentine leukotoxin; enterotoxins (*sea*, *seb*, *see*), accountable for the formation of biofilm (*icaC and bap*) and *spsD*, whose protein binds fibrinogen, fibronectin, and elastin, as well as genes that specifically bind fibrinogen (*fib*), fibronectin (*fnbB*), collagen (*cna*), and sialoprotein (*bbp*).

**Fig. 1:**
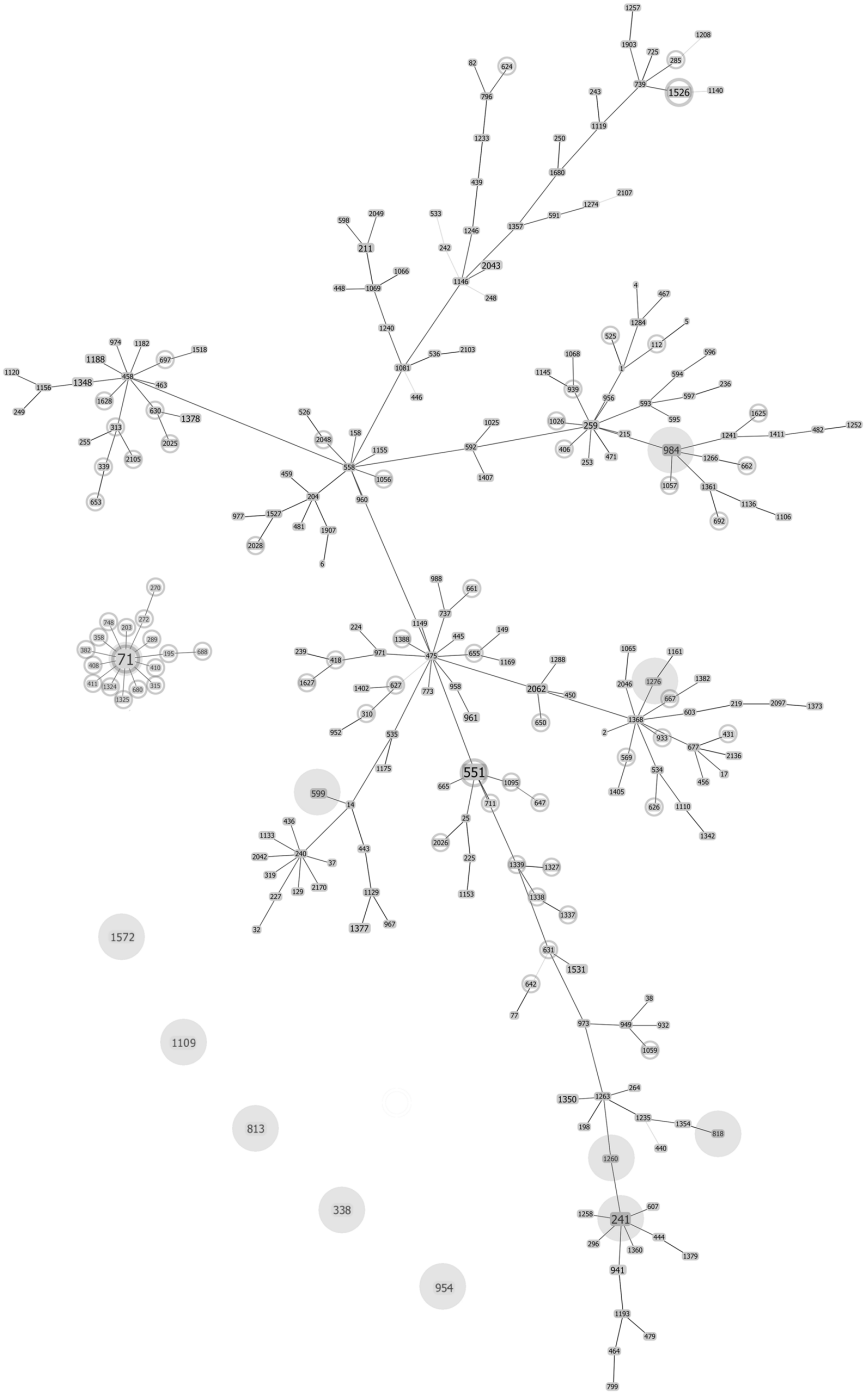
Connection diagram of the *S. pseudintermedius* sequence types found in Europe. Sequence types (STs) identified in this study are signed in gray dot spot areas. STs of methicillin-resistant *S. pseudintermedius* strains are signed in dark gray circles. All remain STs are classified as STs of methicillin-susceptible *S. pseudintermedius* strains

In the isolates used in this study, the *ack* gene sequences had four alleles and four polymorphic sites. Ten polymorphic sites were detected in the *cpn*60 gene, resulting in seven alleles. Sequence analysis of the *fdh* gene revealed three alleles with four polymorphic sites. The *sar* gene sequences had two alleles with one polymorphic site. Six polymorphic sites were detected in the *pta* gene, resulting in five alleles. Eight alleles with six polymorphic sites were obtained in the *purA* gene. Sequence analysis of the *tuf* gene showed one allele. MLST analysis of sequence variation at seven loci showed 12 STs. Two MRSP isolates from animals were classified as the most frequently identified in the database MRSP clone—ST71. None of the STs found in the group of strains isolated from humans occurred in the group of strains isolated from animals. The results of the MLST analysis are shown in Table 3. Figure 1 shows the relationship between the sequence types of the tested isolates and those of European origin reported in the database. The genetic association of the seven obtained STs with the STs reported in Europe was confirmed.

**Table 3:** Sequence type variations and alleles in MLST-7 of *S. pseudintermedius*

Strain no.	Host	Methicillin resistance	ST based on MLST-7	Loci and corresponding alleles						
				*ack*	*cpn*60	*fdh*	*pta*	*purA*	*sar*	*tuf*
SPL1	Human	MSSP	813	1	21	1	2	5	1	1
SPL2	Human	MSSP	241	1	21	4	1	23	1	1
SPL3	Human	MSSP	954	1	2	2	20	18	1	1
SPL4	Human	MSSP	599	5	29	2	1	5	1	1
SPL5	Human	MSSP	1572	1	11	4	75	5	1	1
SPL6	Human	MSSP	984	1	2	2	1	3	2	1
SPL7	Human	MSSP	984	1	2	2	1	3	2	1
SPZ1	Dog	MSSP	818	2	7	4	1	8	1	1
SPZ2	Dog	MSSP	1276	1	2	1	1	8	1	1
SPZ3	Dog	MSSP	338	1	10	2	4	2	1	1
SPZ4	Dog	MRSP	71	3	9	1	2	1	2	1
SPZ5	Dog	MSSP	1109	1	11	4	1	13	1	1
SPZ6	Dog	MSSP	1260	1	2	4	1	23	1	1
SPZ7	Dog	MRSP	71	3	9	1	2	1	2	1

*MRSP* methicillin−resistant *S. pseudintermedius*, *MSSP* methicillin−susceptible *S. pseudintermedius*

## Discussion

The widespread colonization of the mucous membranes and skin of animals by *S. pseudintermedius* means that the transfer of this coagulase-positive species to humans is inevitable (Garbacz et al. 2013; Phumthanakorn et al. 2017; Maali et al. 2018; Malisova et al. 2019). The carrier state of these staphylococci in humans has already been demonstrated (Chrobak et al. 2011; Phumthanakorn et al. 2017). Studies have shown that the pangenome of this species is very wide and includes many invasive factors, which means that the pathogenic potential of these bacteria is high (Garbacz et al. 2013). This observation was confirmed by our studies showing the large variety of isolates obtained in a short period only in laboratories of one hospital and veterinary clinic.

In animals, *S. pseudintermedius* strains most often cause pyoderma and ear infections but are also responsible for systemic diseases (Phumthanakorn et al. 2017; Chrobak-Chmiel et al. 2018). In humans, these staphylococci caused wound infections, sinusitis, soft tissue infections, and endocarditis (Maali et al. 2018; Phumthanakorn et al. 2018). Human infections are not frequently reported, but Maali et al. reported hospital epidemics related to *S. pseudintermedius* (Maali et al. 2018). This information shows that human infections may appear despite the fact that there was no contact with animals. The animal isolates we studied constituted the etiological factor of ear and skin infections. Human strains originated from severe skin lesions and wound infections, and one was isolated from inflammatory lesions in the abdominal cavity (SPL5).

The invasiveness of *S. pseudintermedius* strains described so far concerns mainly the skin. MSCRAMM adhesins are essential in such infections (Phumthanakorn et al. 2017; Maali et al. 2018). These adhesins interact with receptors on keratinocytes, corneocytes, and proteins of the skin’s extracellular matrix (Chrobak-Chmiel et al. 2018). The *sps* genes play an important role in adhesion to the host extracellular matrix (ECM) proteins (Phumthanakorn et al. 2017).

The surface proteins of staphylococcal SpsP and SpsQ, whose genes were present in the strains we studied, are crucial in the adhesion process. These proteins show high homology with *S. aureus* protein A, which binds IgG and inactivates the complement system. These genes were proven to be expressed in *S. pseudintermedius* (Phumthanakorn et al. 2017; Balachandran et al. 2018; Gonzalez-Martin et al. 2020). Our results indicate a tandem arrangement of *spsP* and *spsQ* genes. Phumthanakorn et al. (Phumthanakorn et al. 2017) linked the presence of these genes with the development of skin infections. Genes determining proteins binding the extracellular matrix (ECM), including fibronectin, fibrinogen, and cytokeratin, also present in our strains, may support further invasion of the pathogen (Phumthanakorn et al. 2017; Chrobak-Chmiel et al. 2018; Richards et al. 2018).

Canine keratinocyte invasion is particularly favored by products of the *spsD*, *spsL*, and *spsO* genes (Gonzalez-Martin et al. 2020). The two latter ones were present in the strains we studied. The production of elastin-binding protein promotes the colonization of the host tissue. The gene coding for this protein, *ebpS*, was present in all strains we tested. Most strains also had the *eno* gene, which encodes the enolase, which is a plasminogen receptor, and also determines adherence to laminin, the main component of the basal membrane of the vessels, which allows the spread of infection (Zuniga et al. 2015; Kot et al. 2018).

Adhesion precedes colonization of the infected site and biofilm formation. All the isolates we tested had the *icaD* gene belonging to the icaADCB operon responsible for biofilm formation (Crawford et al. 2016). Admittedly, a biofilm may arise independently of the expression of *icaA* and *icaD* genes. These genes, however, stimulate PIA—the intercellular adhesion polysaccharide—an essential component of the biofilm (Casagrande Proietti et al. 2015; Meroni et al. 2019).

In staphylococci, proteases and lipases are considered important virulence factors (da Cunha et al. 2006; Krzyminska et al. 2015; Haddad et al. 2018). These factors determine the breakdown of sebum, the skin’s protective layer, and promote the spread of bacteria (Kmieciak and Szewczyk 2018). The ability to proteolyze and the gene responsible for lipase production were found in all the isolates we studied.

Coagulase is undoubtedly a vital enzyme in the pathogenicity of *S. pseudintermedius*. It enables bacteria to escape phagocytosis (Chrobak-Chmiel et al. 2018; Sewid et al. 2018). Coagulase was produced by all the isolates we tested. However, only in the strains isolated from humans the clumping factor (CF) was present. In addition to promoting tissue adhesion, this factor causes platelet aggregation and bacterial clumping in the plasma. This protects bacteria against phagocytosis by neutrophils. Its presence is important in cases of septic arthritis and infective endocarditis (Geoghegan et al. 2009; Lacey et al. 2016, 2019).

In *S. aureus*, Panton-Valentine leukocidin (PVL) was associated with severe skin infections (Hoppe et al. 2019). The equivalent of PVL in the *S. pseudintermedius* is the binary leukotoxin Luk-I consisting of the secreted proteins LukF-I and LukS-I (Garbacz et al. 2013; Chrobak-Chmiel et al. 2018). In the tested strains, we found both genes coding these proteins. All isolates also had the *siet* gene encoding the exfoliating toxin. This gene is common in *S. pseudintermedius* (Ruscher et al. 2010; Garbacz et al. 2013; Gharsa et al. 2015; Melter et al. 2017). This toxin is essential in pyoderma and chronic otitis in animals (Bannoehr and Guardabassi 2012; Garbacz et al. 2013; Banovic et al. 2017; Gonzalez-Martin et al. 2020). In the genomes of the tested strains isolated from humans, we also found other genes that determine the presence of exfoliative toxins: *exiA* and *exiB*. These toxins are responsible for the intra-epidermal cleavage caused by the degradation of desmoglein (Bannoehr and Guardabassi 2012; Richards et al. 2018; Gonzalez-Martin et al. 2020).

In some strains isolated from humans, we detected enterotoxin genes. Of the most important enterotoxins A-E (SEA-SEE) sought, these strains encoded SEC or SED toxin, one of them both. These superantigens bind T lymphocytes to MHC molecules and overstimulate them, leading to acute clinical symptoms (Yoon et al. 2010). These toxins are mainly responsible for food poisoning (Piechota et al. 2014). Chrobak-Chmiel et al. (Chrobak-Chmiel et al. 2018) indicated, however, that simultaneous production of exfoliating toxins and enterotoxins may significantly affect the course of pyoderma.

The assessment of antibiotic resistance of the studied strains indicates their broad resistance regarding the antibiotics used in therapy, including in veterinary medicine. Resistance to β-lactam antibiotics reached 79%. Other authors also noted *S. pseudintermedius* resistance to this class of antibiotics (Carroll et al. 2021). This resistance may be due to modified penicillin-binding proteins (*mecA* gene), production of β-lactamases (*blaZ* gene), or tolerance phenomena. Enzymatic resistance of staphylococci to penicillins is widespread (Silva et al. 2021), and our strains often present this resistance. Both methicillin-resistant strains (MRSP) also had the *blaZ* gene and, in addition, proved to be resistant to the greatest number of tested antibiotics—they were isolates from dogs.

The MLST method allowed us to assess the population structure of the strains we collected. Although the isolates collection came from one small region, it was very diverse. Twelve types of ST sequences were obtained, nine of which had previously been registered in Europe. None of the MSSP STs shown by us has been previously reported in Poland. Strains isolated from humans turned out to be different from those from animals, which contradicts the common belief about the animal nature of *S. pseudintermedius* infection. These data show a high level of recombination within the species and, in consequence, its poor clonality. Our animal MRSP isolates belong to the ST71 sequence dominant in Northern Europe. To date, only three such human isolates have been reported. The relationship between the ST71 sequence and other MRSP sequence types ST680 and ST551 present in Poland has already been shown (Bannoehr et al. 2007; Black et al. 2009).

## Conclusion

The animal and human isolates we studied have essential features that allow them to infect both hosts. There are similarities between the groups, but at the same time, the strains that compose them are very diverse, confirming the data on the extent of the pangenome of this species. Important features such as being members of different ST groups, the presence of specific genes, or antibiotic resistance features contradict the often-presented view of the exclusively zoonotic nature of *S. pseudintermedius* infections in humans. The presence of genes determining the severe course of infections makes us recognize the importance of this species and the need for better monitoring of its occurrence.

## Supplementary Information

Below is the link to the electronic supplementary material.

Supplementary file1 (PDF 441 KB)
