# Supplementary material for: Pathogenic potential and antimicrobial resistance of Staphylococcus pseudintermedius isolated from human and animals
Source: Folia Microbiol (Praha). 2022 Oct 12;68(2):231–43. doi: 10.1007/s12223-022-01007-x (PMC10104922; doi:10.1007/s12223-022-01007-x)
Supplement: Supplementary file 1 — Supplementary file1 (PDF 441 KB) [file 12223_2022_1007_MOESM1_ESM.pdf]

Folia Microbiologica

Pathogenic potential and antimicrobial resistance of *Staphylococcus pseudintermedius* isolated from human and animals.

Paulina Glajzner [a], Eligia M. Szewczyk [a], Magdalena Szemraj [a]*

[a] Department of Pharmaceutical Microbiology and Microbiological Diagnostics, Medical University of Lodz, Łódź, Poland, ul. Muszyńskiego 1, 90-001 Łódź, Poland

* Corresponding author: Magdalena Szemraj; tel.: 48 42 679 300, email: magdalena.szemraj@umed.lodz.pl

Paulina Glajzner, email: paulina.glajzner@umed.lodz.pl; ORCID: 0000-0002-7147-679X

Eligia M. Szewczyk, email: eligia.szewczyk@umed.lodz.pl; ORCID: 0000-0001-7148-4063

Magdalena Szemraj, email: magdalena.szemraj@umed.lodz.pl; ORCID: 0000-0002-1986-2220

Table S1. Primer sequences, size of amplicons and PCR reaction conditions.

The role of the gene	The name of the gene	Sequences	Size of the amplicon (bp)	PCR conditions
Identification gene	*nuc*	pse-F: TRGGCAGTAGGATTCGTTAA<br>pse-R: CTTTTGTGCTYCMTTTTGG	926	95 °C 2 min, 30 cycles (95 °C 30 s, 56 °C 35 s, 72 °C 1 min), 72 °C 2 min
Genes responsible for binding the host extracellular matrix	*ebpS*	ebpS-F: AGACGCCACAGAAAAAGA<br>ebpS-R: GCAGATTGACCTTGTTGA	1040	94 °C 2 min 30 s, 30 cycles (94 °C 30 s, 54 °C 30 s, 72 °C 1 min), 72 °C 10 min
	spsO	spsO-F: ACGTCACCTAGTGCTGTTGATT<br>spsO-R: TGCAACTGGCCGTTACAATAA	696	95 °C 3 min, 30 cycles (95 °C 30 s, 55 °C 1 min, 72 °C 4 min), 72 °C 5 min
	spsP	spsP-F: CAAAGCTGAAGCGAAAGCAGAT<br>spsP-R: GCGATACTTACGCCACCCG	366	
	spsQ	spsQ-F: CAGACAAAGGTATGGACAAAGCG<br>spsQ-R: ATTTCGTGGTTTGCTTTAGCTTCT	217	
	spsL	spsL-F: AACTCCAAAGGCCGAAGAAT<br>spsL-R: CCAGCAACAAGAAGGAGAGG	201	
	spsD	spsD-F: TGGTGTAAAAAGCCCTTCAGGTA<br>spsD-R: TTCCCTTCCCCACTTGCATTA	528	
	spsE	spsE-F: TTTCTCGTTTCTGGGCGT<br>spsE-R: GCGTCTTCTGGTTATCGT	1600	94 °C 2 min 30 s, 30 cycles (94 °C 30 s, 54 °C 30 s, 72 °C 60 s), 72 °C 10 min
	fib	fib-F: CTACAACTACAATTGCCGTCAACAG<br>fib-R: GCTCTTGTAAGACCATTTTTCTTCAC	404	94 °C 5 min, 25 cycles (94 °C 1 min, 55 °C 1 min, 72 °C 1 min), 72 °C 10 min
	fnbB	fnbB-F: GTAACAGCTAATGGTCGAATTGATACT<br>fnbB-R: CAAGTTCGATAGGAGTACTATGTTC	524	
	fnbA	fnbA-F: GCGGAGATCAAAGACAA<br>fnbA-R: CCATCTATAGCTGTGTGG	1280	94 °C 5 min, 30 cycles (94 °C 1 min, 50 °C 1 min, 72 °C 1 min), 72 °C 10 min
	cna	cna-F: GTCAAGCAGTTATTAACACCAGAC<br>cna-R: AATCAGTAATTGCACTTTGTCCACTG	423	94 °C 5 min, 25 cycles (94 °C 1 min, 55 °C 1 min, 72 °C 1 min) 72 °C 10 min
	eno	eno-F: ACGTGCAGCAGCTGACT<br>eno-R: CAACAGCATCTTCAGTACCTTC	302	
	bbp	bbp-F: AACTACATCTAGTACTCAACAACAG<br>bbp-R: ATGTGCTTGAATAACACCATCATCT	575	
	coa	coa-F: TTTGGCCATGGATGAAAAAGAAATTGCTT<br>coa-R: TTTGGGGATCCTGACCGTTGTAAGCTTTAT	1500	94 °C 1 min 30 s, 35 cycles (94 °C 1 min, 55 °C 2 min, 72 °C 1 min), 72 °C 5min
Genes responsible for formation of a biofilm	*icaC*	icaC-F: ATGGGACGGATTCCATGAAAAAGA<br>icaC-R: TAATAAGCATTAATGTTCAATT	1100	95 °C 10 min, 25 cycles (94 °C 1 min, 55 °C 1 min, 72 °C 1 min), 72 °C 10 min

	icaD	icaD-F: CGTTAATGCCTTCTTTCTTATTGCG<br>icaD-R: ATTAGCGCACATTCGGTGTT	166	94 °C 3 min, 35 cycles (94 °C 15 s, 56 °C 20 s, 72 °C 20 s), 72°C 5 min
	bap	bap-F: CCCTATATCGAAAGGTGTAGAATTG<br>bap-R: GCTGTTGAAGTTAATACTGTACCTGC	971	94 °C 2 min 30 s, 30 cycles (94 °C 45 s, 62 °C 1 min, 72 °C 1 min), 72 °C 7 min
Genes responsible for production of enterotoxins	*sea*	sea-F: CCTTTGGAAACGGTTAAAACG<br>sea-R: TCTGAACCTTCCCATCAAAAAC	127	95 °C 2 min, 30 cycles (95 °C 1 min, 55 °C 1 min, 72 °C 2 min), 72 °C 5 min
	seb	seb-F: TCGCATCAAACTGACAAACG<br>seb-R: GCAGGTACTCTATAAGTGCCTGC	477	
	sec	sec-F: CTCAAGAACTAGACATAAAAGCTAGG<br>sec-R: TCAAAATCGGATTAACATTATCC	271	95 °C 10 min, 35 cycles (95 °C 30 s, 53 °C 45 s, 72 °C 90 s), 72 °C 10 min
	sed	sed-F: CTAGTTTGGTAATATCTCCTTTAAACG;<br>sed-R: TTAATGCTATATCTTATAGGGTAAACATC	319	95 °C 2 min, 30 cycles (95 °C 1 min, 55 °C 1 min, 72 °C 2 min), 72 °C 5 min
	see	see-F: CAGTACCTATAGATAAAGTTAAAACAAGC<br>see-R: TAACTTACCGTGGACCCTTC	178	
Genes responsible for production exfoliative toxins and leukotoxins	*exiA*	exiA-F: AGTAACAAACTATCACATAGCG<br>exiA-R: TTAACAGGTTATAACGTCCCC	455	94 °C 5 min, 35 cycles (94 °C 30 s, 53 °C 30 s, 72 °C 1 min), 72 °C 5 min
	exiB	exiB-F: AAATTATTTTCACTCCAGCTT<br>exiB-R: CATGTATACCTATTAGTTCCCC	381	
	siet	siet-F: ATGGAAAATTTAGCGGCATCTGG<br>siet-R: CCATTACTTTTCGCTTGTTGTGC	359	94 °C 5 min, 35 cycles (94 °C 30 s, 56 °C 30 s, 72 °C 1 min), 72 °C 5 min
	LukS	LukS-F: CAGGGATCCGCAAACACTATAGAAGAAATCG<br>LukS-R: GCTTGTCGACCTATTAATTATGCCCCTTTAC	868	94 °C 5 min, 25 cycles (94 °C 30 s, 58 °C 30 s, 72 °C 1 min), 72 °C 5 min
	LukF	LukF-F: TGTCGGATCCGCTAATCAAATTACACCTG<br>LukF-R: GTCAGTCGACCTATTATGATGGGTTTTTTTCATC	926	
	pvl	pvl-F:TCATTAGGTAAAATGTCTGGACATGATCCA<br>pvl-R: GCATCA ASTGTATTGGATAGCAAAAGC	433	98 °C 30 s, 30 cycles (98 °C 5 s, 57 °C 10 s, 72 °C 10 s), 72 °C 5 min
Gene responsible for lipase production	*lip*	lip-F: GGAAAAGCAGCAGAAAGAA<br>lip-R: GGGTGCTGTGATGAAATA	1601	94 °C 2 min 30 s, 30 cycles (94 °C 30 s, 54 °C 30 s, 72 °C 1 min), 72 °C 10 min
Genes for MLST analysis	*ack*	ack-F: CACCACTTCACAACCCAGCAAACT<br>ack-R: AACCTTCTAATACACGCGCACGCA	680	95 °C 1 min 30 s, 35 cycles (52 °C 30 s, 72 °C 1 min, 94 °C 30 s, 52 °C 30 s), 72 °C 5 min
	*cpn*60	cpn60-F: GCGACTGTACTTGCACAAGCA<br>cpn60-R: AACTGCAACCGCTGTAAATG	552	
	fdh	fdh-F: TGCGATAACAGGATGTGCTT<br>fdh-R: CTTCTCATGATTCACCGGC	408	
	purA	purA-F: GATTACTTCCAAGGTATGTTT<br>purA-R: TCGATAGAGTTAATAGATAAGTC	490	
	pta	pta-F: GTGCGTATCGTATTACCAGAAGG	570	

		pta-R: GCAGAACCTTTTGTTGAGAAGC		
	sar	sar-F: GGATTTAGTCCAGTTCAAAATTT	521	
		sar-R: GAACCATTCGCCCCATGAA		
	tuf	tuf-F: CAATGCCACAAACTCG	500	
		tuf-R: GCTTCAGCGTAGTCTA		
Antibiotic resistance genes	*mecA*	mecA-F: AAAATCGATGGTAAAGGTTGGC	532	95° 4 min, 30 cycles (95 °C 60 s, 58 °C 60 s, 72 °C 60 s), 72 °C 7 min
		mecA-R: AGTTCTGCAGTACCGGATTTGC		
	blaZ	blaZ-F: TGACCACTTTTATCAGCAAC	700	
		blaZ-R: GCCATTTCAACACCTTCTTTC		
